# Clinical significance of prophylactic central compartment neck dissection in the treatment of clinically node-negative papillary thyroid cancer patients

**DOI:** 10.1186/s12957-016-1003-5

**Published:** 2016-09-19

**Authors:** Claudio Gambardella, Ernesto Tartaglia, Anna Nunziata, Graziella Izzo, Giuseppe Siciliano, Fabio Cavallo, Claudio Mauriello, Salvatore Napolitano, Guglielmo Thomas, Domenico Testa, Gianluca Rossetti, Alessandro Sanguinetti, Nicola Avenia, Giovanni Conzo

**Affiliations:** 1 Department of Anaesthesiology, Surgery and Emergency Sciences, Second University of Naples, Via Pansini 5, 80131 Naples, Italy; 2 Endocrine Surgical Unit, University of Perugia, Perugia, Italy

**Keywords:** Total thyroidectomy, Papillary thyroid cancer, Prophylactic lymph node dissection, Routine central lymph node dissection, Therapeutic lymph node dissection, Radioactive iodine ablation

## Abstract

**Background:**

Lymph nodal involvement is very common in differentiated thyroid cancer, and in addition, cervical lymph node micrometastases are observed in up to 80 % of papillary thyroid cancers. During the last decades, the role of routine central lymph node dissection (RCLD) in the treatment of papillary thyroid cancer (PTC) has been an object of research, and it is now still controversial. Nevertheless, many scientific societies and referral authors have definitely stated that even if in expert hands, RCLD is not associated to higher morbidity; it should be indicated only in selected cases.

**Main body:**

In order to better analyze the current role of prophylactic neck dissection in the surgical treatment of papillary thyroid cancers, an analysis of the most recent literature data was performed. Prophylactic or therapeutic lymph node dissection, selective, lateral or central lymph node dissection, modified radical neck dissection, and papillary thyroid cancer were used by the authors as keywords performing a PubMed database research. Literature reviews, PTCs large clinical series and the most recent guidelines of different referral endocrine societies, inhering neck dissection for papillary thyroid cancers, were also specifically evaluated. A higher PTC incidence was nowadays reported in differentiated thyroid cancer (DTC) clinical series. In addition, ultrasound guided fine-needle aspiration citology allowed a more precocious diagnosis in the early phases of disease. The role of prophylactic neck dissection in papillary thyroid cancer management remains controversial especially regarding indications, approach, and surgical extension. Even if morbidity rates seem to be similar to those reported after total thyroidectomy alone, RCLD impact on local recurrence and long-term survival is still a matter of research. Nevertheless, only a selective use in high-risk cases is supported by more and more scientific data.

**Conclusions:**

In the last years, higher papillary thyroid cancer incidence and more precocious diagnoses were worldwide reported. Among endocrine and neck surgeons, there is agreement about indications to prophylactic treatment of node-negative “high-risk” patients. A recent trend toward RCLD avoiding radioactive treatment is still debated, but nevertheless, prophylactic dissections in low-risk cases should be avoided. Prospective randomized trials are needed to evaluate the benefits of different approaches and allow to drawn definitive conclusions.

## Background

Papillary thyroid cancer (PTC) is the most common endocrine cancer, and during the last decades, its incidence has worldwide increased, more than 240 % in the USA in 2012. In PTCs localized to the thyroid gland, an excellent survival is still reported with 5-year survival rates >99 %. Lymph nodal involvement is very common, and in up to 80 % of cases, lymph node micrometastases are observed [1, 2]. Positive regional lymph nodes decrease 5-year survival rates to 97 %, and in most cases is associated to higher regional recurrence rates [3]. Nevertheless, the impact of lymph node involvement on local recurrence and survival is subject of research trials [4–7]. Moreover, in the last decades, thanks to a better sensitivity of cancer detection methods, more efficacy of diagnostic imaging and molecular markers, a precocious diagnosis allowed to identify a higher number of node-negative low-risk patients, in which the mean tumor size was mostly less than 2 cm, as well as a sensible increase of microcarcinoma incidence was reported in different series. Better outcomes, a favorable clinical evolution, a reduced locoregional recurrence rate, and a prolonged survival are expected in these cases, and consequently, a “tailored” and “less aggressive” management should be suggested to avoid an expensive and risky “overtreatment”. However, according to more recent literature data, in high-risk patients, lymphatic metastases might affect survival, significantly increasing locoregional relapse rate, while in low-risk cases they do not modify the long-term outcomes [8].

The role of total thyroidectomy (TT) remains well-established, while recently, a new substantial agreement exists among endocrinologists regarding thyroid stimulating hormone (TSH) suppression therapy and postoperative radioactive iodine (RAI) treatment [9].

A “low aggressive behavior” in most PTC patients, a low locoregional recurrence rate, often an irrelevant mortality, associated with a need of long-term follow-up (more than 20 years), makes difficult the indications to routine central lymph node dissection (RCLD), in the absence of an enlarged lymph node (preoperative ultrasound and intraoperative inspection) in PTC ranging between 1 and 2 cm of diameter. Considering the high risk of positive lymph nodes, better outcomes and a lower morbidity rate associated with the first operation RCLD is suggested, even if supposed higher morbidity rates are advocated by some authors, in the absence of any demonstrable benefits in terms of long-term survival [7]. In the management of differentiated thyroid cancer (DTC), a routine lymph node dissection may be selectively suggested with similar oncologic outcomes reported after more extensive and risky dissections even if the literature review demonstrated that indications to RCLD or to RAI ablation, in PTC ranging between 1 and 2 cm of diameter, are a matter of intensive research [10, 11]. According to the revised American Thyroid Association (ATA) guidelines (2009), prophylactic dissection in clinical node-negative patients, generally followed by lower postoperative thyroglobulin (Tg) serum levels, may prevent a future recurrence, allowing a lower morbidity rate than a second surgery [9]. Nevertheless, even if a lower local recurrence is generally advocated, statistically significant benefits in terms of long-term survival and definitive evidence of improved recurrence rates compared to TT alone were not demonstrated [5, 7, 10]. In an attempt to better clarify the current role of RCLD in treating PTC and more intensively investigate its suitable indications, we analyzed the most recent literature data.

### Review section

By using as keywords lymph node dissection, selective, lateral or central lymph node dissection, modified radical neck dissection, prophylactic or therapeutic lymph node dissection, and papillary thyroid cancer, a PubMed database research was carried out limiting our search to English language literature. Prospective, retrospective, and meta-analysis studies were analyzed and in addition, some older articles were considered. The most recent guidelines regarding neck dissection for papillary thyroid cancer, according to the American Thyroid Association (ATA), European Thyroid Association (ETA), Unità operative di Endocrinochirurgia (UEC), American Head and Neck Society, and the American Academy of Otolaryngology-Head and Neck Surgery, were also reported. Regarding terminology of cervical lymphatic anatomy (neck levels) and classification of neck dissection, the most recent ATA guidelines were considered [9, 12]. Lymph node dissection benefits, complications, and impact on locoregional recurrence rate and mortality were evaluated.

## Discussion

DTC is an uncommon neoplasm, representing 2.5 % of all malignancies, with an increasing mean annual incidence per 100.000 individuals ranging from 1.2 to 2.6 in men and from 2.0 to 3.8 in women [13]. It is the fifth most common cancer in women in the USA [1, 4]. In the last decades, PTC incidence has increased more than 240 %, and moreover, US-guided fine-needle aspiration citology (FNAC) allowed a more precocious diagnosis. Papillary variants are by far the most frequent neoplasms, followed by follicular (10 to 20 % of cases) and medullary thyroid cancers (5 to 8 % of cases), often part of MEN2 syndrome [14, 15]. Anaplastic thyroid carcinoma represents a very rare and aggressive thyroid neoplasm that belongs to the group of killer tumors, with a mean survival period of 6.2 months, as reported for carcinosarcoma of other districts [16, 17]. Differently from the past, a higher number of small papillary cancers (<1 cm) and a more precocious diagnosis are reported in clinical series requiring a less aggressive multimodal treatment [18, 19]. As a matter of fact, in most PTC patients, prognosis is more and more favorable and is associated with a 10-year mortality rate of about 7 % [20, 21]. However, about 20 % of patients face the morbidity of locoregional recurrences, and PTC-related deaths [22–24]. Macroscopic lymph node involvement of central and lateral levels is very common in PTC, and micrometastases are observed in up to 80 % of cases, especially in young patients [18]. Even if some authors retain that they may be responsible for thyroglobulin higher postoperative serum level, micrometastases do not affect the clinical course of most PTC patients. Infiltration of thyroid capsule, patient age (pediatric or geriatric population), tumor size, and several oncogenes (p53, BRAF) are associated with node involvement, representing the main risk factors for recurrence [25]. Nevertheless, no clinical or pathological factors might certainly predict lymph node metastases. The observed discordance between the high rate of lymph node micrometastases, and the low incidence of clinical recurrence following TT without RCLD, may be correlated to postoperative RAI administration but testifies the indolent PTC nature. Nevertheless, recently, it has been hypothesized that, especially in older patients, lymphatic metastases may affect recurrence and survival rates. In the treatment of low-risk clinically node-negative DTC patients with a cancer exceeding 10 mm of diameter, TT should be considered as the operation of choice, as in most thyroid diseases [26–28]. In addition, there is agreement about RCLD indications in high-risk patients—defined as male patient, age >45 years, *T* > 3 cm, and BRAF-positive. On the contrary, the role of RCLD in low-risk cases is to date under investigation, and endocrine and neck surgeons are divided between pros and cons. According to its proponents, a better chance of cure, reducing the recurrence risk, may be achieved with a low morbidity. Moreover, the high incidence of lymph node metastases, the observation that reoperation for central recurrence may have a greater morbidity, the insufficient diagnostic accuracy of intraoperative inspection and of ultrasonography, reported in 1/3 of DTC patients, and the failure of ^131^I ablation in about 30 % of cases, are considered in favor of RCLD. The procedure allows a better staging too, but a prospective randomized study of RCLD could be very expensive and not readily feasible [29]. ATA guidelines, published in 2006, stated that RCLD should be considered in DTC, but this recommendation is not based on strong supporting data [30]. Recently, according to ATA and UEC, prophylactic dissection could be especially undertaken in high-risk patients with advanced primary tumors, so recognizing that this approach may be associated with increased morbidity, especially among low-volume surgeons [9, 31]. Rates of permanent hypoparathyroidism and of unintentional permanent recurrent laryngeal nerve injury after TT were, respectively, 1–2 and 0–5.5 %, whereas following TT associated with RCLD, they, respectively, increased to 0–14.3, and to 0–5.7 %, according to ML White et al. [32]. The author concluded that central nodal dissection reduces locoregional recurrence, improves disease-free survival, and increases the number of patients with undetectable Tg levels, although is associated with a higher risk of injury to parathyroid glands. In case of unilateral cancer, with the aim to reduce RCLD morbidity, some authors, as alternative approach, proposed ipsilateral procedure with variable results [33]. This recent proposal appears to be very interesting, but conclusive data have still not been reported. Pacini et Al., stated that even if associated to a better staging, RCLD does not offer yet further benefits [34]. A prospective randomized study of RCLD is very expensive and not readily feasible and so, without sufficient statistical power to demonstrate significant differences in outcomes, the role of prophylactic surgery is still to be demonstrated [29]. A better cancer staging and sensible reduction of postoperative serum Tg levels is really expected, but prospective randomized trials are needed to evaluate the benefits of prophylactic dissection [35]. TT, followed by RAI administration and TSH suppression therapy, may guarantee optimal long-term results, with a low incidence of locoregional lymph node recurrence [8]. Regarding reoperation (lymph node dissection) outcomes, a higher morbidity is not reported especially following unilateral procedures [5]. Nevertheless, RCLD seems to have a role in DTC staging and is useful to modify the treatment protocol. Travagli et al. reported a 30 % increase in the number of patients with T1 DTC (preoperatively considered node-negative), for whom ^131^I ablation was indicated following routine central dissection, demonstrating unexpected nodal metastases [25]. In the absence of an enlarged lymph node, and especially when RAI administration is advisable, routine lymph node dissection is not indicated [36–38]. Moreover some authors reported a significative incidence of lateral lymph node relapse that was unaffected by routine central dissection [39]. On the other hand, in low-risk patients with tumors ≤1 cm, lymph node dissection may however discover metastases requiring RAI ablation.

## Conclusions

The role of routine neck dissection remains a matter of research, and the frequent lateral postoperative involvement might be cited against its supposed benefits, avoiding risky morbidity.

In the absence of data supporting the favorable effects of RCLD, we believe that, in the treatment of PTC without a suspicious enlarged lymph node, it is not indicated, and more prospective, randomized controlled studies with large sample and sufficient follow-up are needed in the attempt to better define its clinical significance and demonstrate its prognostic impact. In addition, in identifying high-risk patients, more accurate and definite criteria might be investigated for a better preoperative assessment and a tailored surgery.

## Abbreviations

ATA: American Thyroid Association
DTC: differentiated thyroid cancer
ETA: European Thyroid Association
FNAC: fine-needle aspiration citology
PTCs: papillary thyroid cancers
RAI: radioactive iodine
RCLD: routine central lymph node dissection
TSH: thyroid stimulating hormone
TT: total thyroidectomy
UEC: Unità operative di Endocrinochirurgia
